# Colectomy Complicated by High-Output Ileostomy Managed in a Virtual Hybrid Hospital-at-Home Program

**DOI:** 10.1155/2022/3177934

**Published:** 2022-09-29

**Authors:** Margaret R. Paulson, Karla Maita, Francisco R. Avila, Ricardo A. Torres-Guzman, John P. Garcia, Abdullah Eldaly, Antonio J. Forte, Michael J. Maniaci

**Affiliations:** ^1^Division of Hospital Internal Medicine, Mayo Clinic Health Systems, Eau Claire, Wisconsin, 2321 Stout Road, Menomonie, Wisconsin 54751, USA; ^2^Division of Plastic Surgery, Mayo Clinic, Jacksonville, Florida, 4500 San Pablo Road, Jacksonville, Florida 32224, USA; ^3^Division of Hospital Internal Medicine, Mayo Clinic, Jacksonville, Florida, 4500 San Pablo Road, Jacksonville, Florida 32224, USA

## Abstract

Chronically ill patients with superimposed acute illness requiring hospitalization are more likely to develop an extended length of stay, hospital-acquired infections, and adverse events throughout their hospitalization. An excellent alternative to managing this population of patients in the traditional bricks-and-mortal (BAM) hospital is the hospital-at-home (HaH) model. The Advanced Care at Home (ACH) program is Mayo Clinic's HaH model that provides acute and postacute care to high-acuity patients in their homes rather than in the traditional hospital and skilled nursing facility. We report a case of postoperative care through the ACH program of a patient suffering from short gut syndrome, high-output ileostomy, and severe protein-calorie malnutrition in the setting of previously diagnosed triple-negative invasive ductal carcinoma (IDC) of the right breast complicated by lung and brain metastasis. The patient had multiple complications that required repeated scare escalations directed by a multidisciplinary virtual care. Despite these complications, the ACH model of care was able to keep the patient in the home setting the majority of the time, limiting BAM hospital days, and eliminating the need to use the emergency department for acute escalation for 3 months. The patient was able to recover during this time period and proceed to successful take-down of the ileostomy. This case highlights the benefits of the ACH program by offering high-acuity hospital-level care to severely ill patients in the comfort of their homes. Highly qualified providers paired with curated technology in the home allowed for prompt identification of patient decompensation and timely initiation of treatment while avoiding institutionalization.

## 1. Introduction

In the United States, the number of visits to the emergency department (ED) has steadily increased each year [1]. Similarly, the United Kingdom population has described an increase in more complex cases going to the ED, especially patients over 65 years with more than five underlying conditions, challenging management [2]. This increased volume of complex patients in the ED setting can lead to extended use of ED resources, increased hospital readmission rates, and a longer inpatient length of stay (LOS), putting patients at higher risk for hospital-acquired infections, delirium, and deconditioning [1, 3–5].

Mayo Clinic's hospital-at-home program (HaH), Advanced Care at Home (ACH), provides acute and postacute care to high-acuity patients in their homes rather than in the traditional hospital and skilled nursing facility [6, 7]. These virtual innovative, curated technology patients are monitored by qualified professionals located in a command center who partner with team members in the patient's home. Our ACH model includes a postacute phase of care following hospitalization, which has previously been shown to decrease hospital readmissions, ED visits, and skilled nursing facility admissions [8]. This is particularly relevant for patients with multiple comorbidities already at risk for increased healthcare utilization and readmission. Implementing this innovative model mitigates the increasing demand for traditional hospital beds and the negative aspects associated with lengthy and recurrent hospitalizations [9–12]. Here, we report the acute and postacute care of a severely ill patient suffering from short gut syndrome with a high ileostomy output and severe protein-calorie malnutrition successfully managed at home through the Mayo Clinic's ACH program. The CARE guidelines for case reports were followed [13].

## 2. Case Report

A 64-year-old lady with a history of metastatic breast cancer presented December 30, 2021, to the ED with severe abdominal pain, distension, vomiting, and nausea to the emergency department in. She had a history of grade 3 invasive ductal carcinoma (IDC) of the right breast diagnosed in March 2017. She was treated with for 6 months with adjuvant chemotherapy (doxorubicin hydrochloride, cyclophosphamide, and paclitaxel), subsequent bilateral mastectomy, and postsurgical radiation therapy. Ten months later, she was found to have radiographic evidence of recurrence with lung and brain metastasis. She underwent chest wall radiation and gamma knife radiation therapy with subsequent immunotherapy with pembrolizumab and binimetinib. These immunotherapy treatments continued until October 2021 when she developed proximal ileum enteritis believed to be due to the immunotherapy. She was managed with symptomatic care until her admission on December 30, 2021. Additional past medical history included essential hypertension complicated by heart failure with preserved ejection fraction, rheumatoid arthritis, chronic anemia, obstructive sleep apnea, radiation-related pulmonary fibrosis, overactive bladder, gastrointestinal reflux, generalized anxiety disorder, chronic migraine, glaucoma, and left iliac artery.

**Figure 1:**
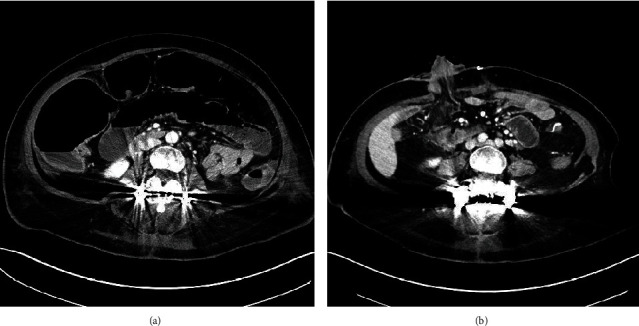
Pre- and postoperative abdominal CT scan. (a) Dilation of multiple small bowel and colon loops, consistent with ileus. Development of right colonic pneumatosis and multiple locules of free intraperitoneal air concerning of underlying perforation. (b) Interval decrease in subcutaneous fluid collection underlying the incision. No evidence for intraperitoneal or abdominal wall fluid collections. Stable to mildly increased stranding in the mesentery underlying the stoma may represent postoperative changes or developing fat necrosis.

In the ED, medical evaluation and imaging revealed acute enterocolitis with intestinal perforation. The colorectal surgery service was consulted, and she underwent an exploratory laparotomy which revealed ischemic enterocolitis of 80 cm of midileum, ascending, and transverse colon with cecum perforation. Right colectomy and resection of the mid ileum with end ileostomy was performed. In discontinuity with the rest of the small bowel, other portion of the end terminal ileum was exteriorized as a mucous fistula (Figure 1). No complications were reported, and she was admitted to the BAM hospital for postoperative care.

The patient was discharged home on postoperative day 9 but returned later the same day presenting with intense abdominal pain. After evaluation, she was readmitted with the diagnosis of intractable pain and abdominal wall seroma, which was immediately drained. A palliative care consultation was required for pain management. This readmission was complicated by the persistency of high output through the ileostomy (>2 L/day). The patient was informed that she would require an extended hospital stay in order to address the high ostomy output, including repeated intravenous (IV) fluid infusions for volume replacement, IV and oral electrolyte replacement, and at least daily laboratory studies. She was also informed to expect a long and complicated postacute course with the risk of future readmissions due both her current condition and complicated, multiple medical comorbidities. The patient inquired if there were any alternative care pathways in order to improve her quality of life and achieve her goal of getting home. The ACH program was described in detail to her, and because she met the clinical and social criteria [6], she was a candidate to receive her hospital care at home. The patient decided to proceed with this alternate care option, and she was consented and accepted into the program.

On January 11, 2022, she transferred to the ACH program acute phase for continued management of high-output ileostomy, and protein-calorie malnutrition. Upon transfer to ACH, she received hybrid virtual management with a combination of daily virtual and in-person visits. A physician or an advanced practice provider (APP) performed a virtual interview from the command center, and in-person team members in home executed the physical exam. Management focused on achieving ileostomy output of less than 1.5 L/day, as recommended by the surgical team. Initially, a high ileostomy output protocol was started with psyllium, loperamide, and diphenoxylate-atropine. In addition to the ACH providers, a multidisciplinary team was involved in the patient's care. This included colorectal surgery, gastroenterology, nephrology, urology, oncology, psychiatry, pharmacy, wound care, nutrition, palliative care, and physical therapy. The chronic comorbidities of the patient required her to be on multiple medications and on total parenteral nutrition (TPN).

**Figure 2:**
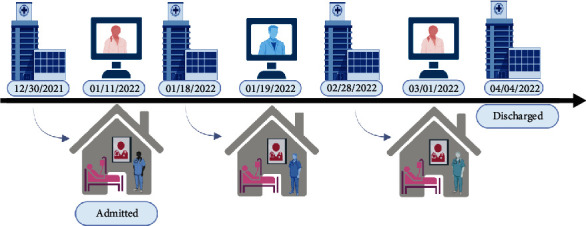
Timeline of intervention and outcomes. Admissions and discharge dates to the B&M hospital and the ACH program in Mayo Clinic Florida, created with BioRender.

On January 18, 2022, she experienced an episode of acute decompensation, characterized by altered mentation. Her ACH physician conducted a virtual evaluation while an in-home APP conducted a thorough physical exam. It was determined that she would need advanced imaging of the brain to rule out new acute pathology. As this could not be done in the home setting, she was transferred directly to the radiology department in the BAM hospital, and a brain MRI was conducted which showed the absence of organic causes and confirmed no evidence of new metastatic lesions. Pain medication polypharmacy (oxycodone and fentanyl patch) was the attributed cause of the mental status change, and once it was determined, no further imaging or invasive procedures were necessary; she was transported back home with ACH on January 19, 2022 (Figure 2).

By early February 2022, after multiple changes in stool medication regimen, the patient had significant improvement in the ileostomy output, with stool output decreased to 1.2 to 1.5 L/day, as well as improved stool consistency. The patient was maintained in the acute phase of care as the patient continued to meet status criteria for inpatient level of care.

On February 27, 2022, she noticed a dramatic increase in the ileostomy output associated with dizziness. A virtual evaluation was conducted by the command center physician aided by an in-home registered nurse who reported orthostatic hypotension on exam. She was given an IV fluid bolus in the home, and in-home labs revealed an acute rise in creatinine indicating acute kidney injury. The ACH virtual medical team discussed the case with the inpatient gastroenterology service who first conducted a virtual evaluation and then asked the ACH team to transport the patient back to the BAM for advanced examination and possible endoscopy. The patient was transported directly to a hospital ward bed, again bypassing the need for ED care, on February 28, 2022. Here, a thorough gastroenterology evaluation concluded symptoms were associated with secondary small intestinal bacterial overgrowth. Antibiotic therapy was added, diphenoxylate and atropine dose was increased, and octreotide 50 mg subcutaneously three times a day was initiated. She was maintained in the BAM hospital for 24 hours and then was returned home in the ACH program on March 1, 2022, to complete the updated inpatient care plan.

**Figure 3:**
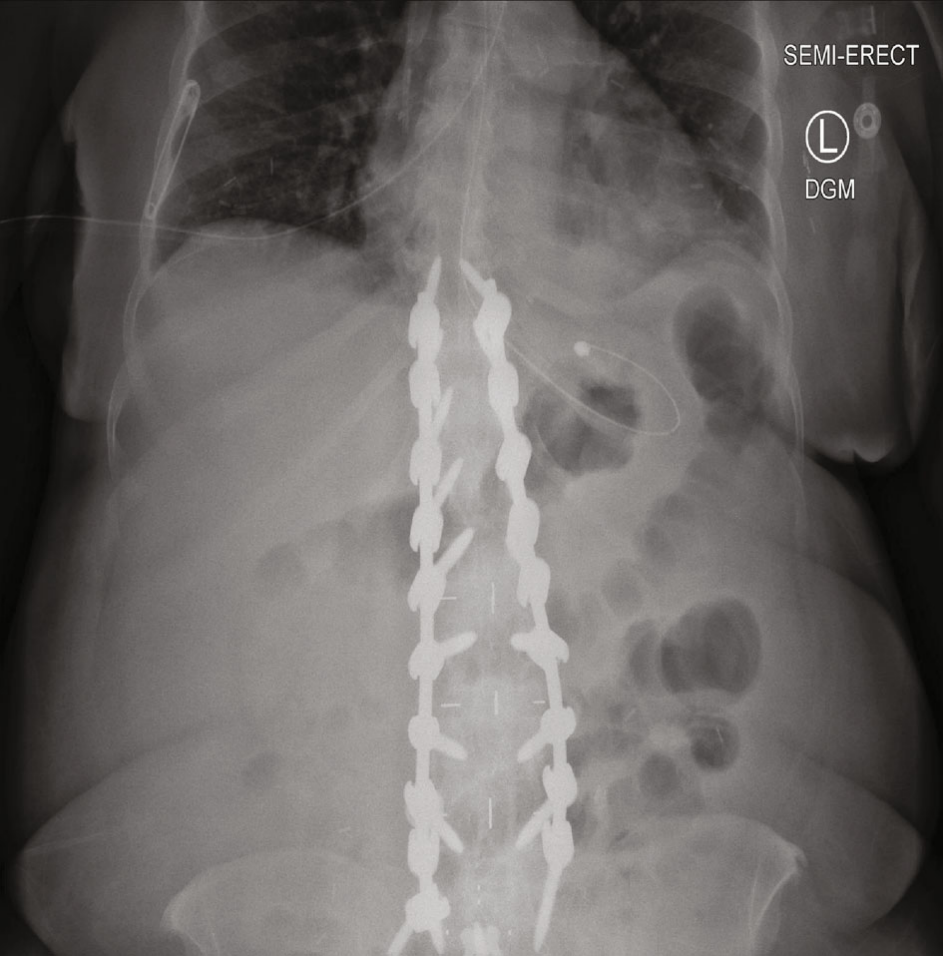
Postoperative abdominal X-ray. Bowel gas pattern is nonobstructive and postsurgical changes from spinal fusion.

She remained in the ACH program until April 4, 2022, moving between the acute care phase and the postacute restorative phase during that time. She remained clinically stable, improved her mobility, and increased her nutritional status while on TPN. On April 6, 2022, after three months under the ACH care, the patient was discharged from the program and admitted to the BAM hospital to undergo a laparotomy to restore the intestinal transit carrying out a side-to-side isoperistaltic ileocolonic anastomosis and side-to-side entero-enterostomy. No complications were reported, and on postoperative day 7, she was discharged from the hospital to home due to a good clinical evolution. A postsurgical abdominal X-ray image was done with evidence of nonobstructive pattern as shown in Figure 3.

## 3. Discussion

This case displays an advanced cancer patient who undergoes intestinal surgery and incurs multiple medical complications over an extended but set period of time. The acute management of a complicated patient in a HaH program requires timely and effective access to hospital-level interventions and highly qualified clinical decision-makers to identify patient decompensation promptly, according to the UK HaH Society [10]. This case report demonstrates how Mayo Clinic's ACH program meets these key characteristics.

Chronically ill and advanced cancer patients represent a challenge for the healthcare system. A group of patients called “superutilizers” are few in number but use a disproportionate amount of healthcare resources [14]. The HaH model is uniquely poised to offer patient-centered care to individuals with complex healthcare needs. HaH allows the patient and care team time to address polypharmacy and avoid excess diagnostic testing and costly ED visits while freeing up hospital bed space for patients who require traditional hospitalization [10, 15]. In our case, the patient was discharged after surgery on January 11, 2022, readmitted the same day, then had continued inpatient-level care through March of 2022 followed by moderate level postacute care through the beginning of April 2022. This period covered 84 days, yet the patient was in the physical BAM hospital space less than 72 total hours once moved into the ACH program: once for advanced imaging with MRI January 18-19 and once for advanced GI examination February 28-March 1. This shows the incredible impact ACH has on BAM inpatient days.

Other research has reported successful application of the HaH model on select populations of acutely ill patients. Heart failure, infections, airway pathologies, and end-of-life cases have been effectively treated through this model of care [16, 17]. This case report adds to that by showing how a hybrid virtual care model could be used to successfully follow up an acutely ill patient with several underlying pathologies. One cause of patient readmission to the BAM hospital was dehydration which has been widely associated with small bowel ostomies [18]. This increased fluid loss through the ostomy has been correlated with the development of acute kidney injury causing a 17% 30-day readmission rate [19]. Proper management prevents the progression to chronic renal disease ending in dialysis. A critical factor in controlling the ostomy output is the patient's diet. For that reason, involving a multidisciplinary team in the care of this patient is essential [20].

Another critical finding in this case is that the ACH program not only reduced BAM hospital days but it also decreased the use of our ED for escalation. During the two escalations reported, assessment of the patient was made by the combination of the command center virtual physician and in in-home APP or nurse. Both times, the patient was able to move directly to the radiology department or the medical wards and bypass the ED, thus conserving valuable ED resources through the coordinated efforts of the ACH and subspecialty care teams. Severely ill patients dealing with multiple comorbidities may require simultaneous evaluation by different specialists working together. Well-developed care coordination allowed multispecialty interaction throughout the course of care. The meticulous daily evaluation by a trained team acutely identified life-threatening situations which were promptly treated. Therefore, implementing this model for superutilizers may decrease the hospital burden and diminish the readmission rate while addressing patient depression and anxiety, as demonstrated in randomized clinical trials studies compared to conventional in-hospital care [15]. Additionally, the evidence of iatrogenic damages related to hospital admission, specifically in the elderly population, supports the implementation of this model of care in the management of chronically ill patients [10].

The quality of the healthcare system can be measured by patients' satisfaction levels [21]. Patients with terminal diseases have been shown to prefer to stay at home and eventually die at home [22]. Additionally, the family support and quality of the healthcare offered at home are other determining factors in the patient's decision to stay at home for palliative care [23]. In this case, the patient's preference to stay at her home demonstrated her satisfaction with the program. While we are unable to report individual quantitative patient satisfaction survey data, the patient affirmed her gratitude and satisfaction of the ACH program on numerous occasions to the ACH staff.

All of these points support the ACH program's efficacy in severely ill patient management. This patient received necessary healthcare for her recovery in the comfort of her home surrounded by her relatives. Furthermore, the ACH team appropriately addressed the challenge of managing acute decompensation. In addition, effective multispecialty consultation and successful polypharmacy mitigation were accomplished.

## 4. Conclusion

High-acuity hospital-level care can be achieved through the Mayo Clinic's ACH model. HaH offers a patient-centered solution to challenges seen with superutilizers of healthcare resources. An individualized care plan created by ACH providers addresses the needs of patients with multiple, complex comorbidities including cancer. A curated technology platform paired with a responsive supplier network and capable in-home care providers allows accurate diagnosis of urgent and life-threatening conditions with implementation of prompt treatment, resulting in both decreased utilization of ED resources as well as decreased time in the brick-and-mortar hospital.

## Data Availability

Data are available upon request.

## References

[1] Andriotti T., Dalton M. K., Jarman M. P. (2021). Super-utilization of the emergency department in a universally insured population. *Military Medicine*.

[2] Wittenberg R., Sharpin L., McCormick B., Hurst J. (2017). The ageing society and emergency hospital admissions. *Health Policy*.

[3] Cotogni P., de Luca A., Evangelista A. (2017). A simplified screening tool to identify seriously ill patients in the emergency department for referral to a palliative care team. *Minerva Anestesiologica*.

[4] Covinsky K. E., Palmer R. M., Fortinsky R. H. (2003). Loss of independence in activities of daily living in older adults hospitalized with medical illnesses: increased vulnerability with age. *Journal of the American Geriatrics Society*.

[5] Krumholz H. M. (2013). Post-hospital syndrome--an acquired, transient condition of generalized risk. *The New England Journal of Medicine*.

[6] Maniaci M. J., Torres-Guzman R. A., Garcia J. P. (2022). Overall patient experience with a virtual hybrid hospital at home program. *SAGE Open Medicine*.

[7] Maniaci M. J., Maita K., Torres-Guzman R. A. (2022). Provider evaluation of a novel virtual hybrid hospital at home model. *International Journal of General Medicine*.

[8] Federman A. D., Soones T., DeCherrie L. V., Leff B., Siu A. L. (2018). Association of a bundled hospital-at-home and 30-day postacute transitional care program with clinical outcomes and patient experiences. *JAMA Internal Medicine*.

[9] Caplan G. A., Sulaiman N. S., Mangin D. A., Aimonino Ricauda N., Wilson A. D., Barclay L. (2012). A meta-analysis of “hospital in the home”. *The Medical Journal of Australia*.

[10] Knight T., Lasserson D. (2022). Hospital at home for acute medical illness: the 21st century acute medical unit for a changing population. *Journal of Internal Medicine*.

[11] Levine D. M., Ouchi K., Blanchfield B. (2020). Hospital-level care at home for acutely ill adults. *Annals of Internal Medicine*.

[12] Dawson N. L., Hull B. P., Vijapura P. (2021). Home telemonitoring to reduce readmission of high-risk patients: a modified intention-to-treat randomized clinical trial. *Journal of General Internal Medicine*.

[13] Riley D. S., Barber M. S., Kienle G. S. (2017). CARE guidelines for case reports: explanation and elaboration document. *Journal of Clinical Epidemiology*.

[14] Mercer T., Bae J., Kipnes J., Velazquez M., Thomas S., Setji N. (2015). The highest utilizers of care: individualized care plans to coordinate care, improve healthcare service utilization, and reduce costs at an academic tertiary care center. *Journal of Hospital Medicine*.

[15] Arsenault-Lapierre G., Henein M., Gaid D., Le Berre M., Gore G., Vedel I. (2021). Hospital-at-home interventions vs in-hospital stay for patients with chronic disease who present to the emergency department. *JAMA Network Open*.

[16] Tibaldi V., Isaia G., Scarafiotti C. (2009). Hospital at home for elderly patients with acute decompensation of chronic heart failure: a prospective randomized controlled trial. *Archives of Internal Medicine*.

[17] Shepperd S., Doll H., Angus R. M. (2009). Avoiding hospital admission through provision of hospital care at home: a systematic review and meta-analysis of individual patient data. *CMAJ*.

[18] Nightingale J. M. D. (2022). How to manage a high-output stoma. *Frontline Gastroenterology*.

[19] Paquette I. M., Solan P., Rafferty J. F., Ferguson M. A., Davis B. R. (2013). Readmission for dehydration or renal failure after ileostomy creation. *Diseases of the Colon and Rectum*.

[20] Budd J., Mafrici B. (2022). Renal dietary management of a patient with a high-output ileostomy and kidney disease: a case study. *Journal of Renal Nutrition*.

[21] Obucina M., Harris N., Fitzgerald J. A. (2018). The application of triple aim framework in the context of primary healthcare: a systematic literature review. *Health Policy*.

[22] Gomes B., Calanzani N., Gysels M., Hall S., Higginson I. J. (2013). Heterogeneity and changes in preferences for dying at home: a systematic review. *BMC Palliative Care*.

[23] Danielsen B. V., Sand A. M., Rosland J. H., Førland O. (2018). Experiences and challenges of home care nurses and general practitioners in home-based palliative care - a qualitative study. *BMC Palliative Care*.

